# Enhancing chronic disease medicine access through public–private collaboration: insights from community pharmacists

**DOI:** 10.3389/fpubh.2026.1839372

**Published:** 2026-06-04

**Authors:** Pengyeow Loh, Kenneth Kwing-Chin Lee, Renukha Sellappans

**Affiliations:** 1 School of Pharmacy, Faculty of Health and Medical Sciences, Taylor's University, Subang Jaya, Selangor, Malaysia; 2 Jeffrey Cheah School of Medicine and Health Sciences, Monash University Malaysia, Subang Jaya, Selangor, Malaysia

**Keywords:** chronic diseases, community pharmacist, community pharmacy, public-private partnership, repeat medicines

## Abstract

**Introduction:**

Patients with chronic diseases in Malaysia frequently return to public health facilities for their monthly medication supply, contributing to congestion and increased workload. At the same time, community pharmacists remain underutilized due to limited dispensing opportunities. This study explored community pharmacists’ perspectives on participating in a public–private partnership model to supply repeat medicines to patients from public health facilities.

**Methods:**

A concurrent embedded mixed-methods design was employed, combining a nationwide quantitative survey of 433 community pharmacists with in-depth interviews involving 15 pharmacists from different states. Quantitative data were analyzed using descriptive and inferential statistics, while qualitative interviews underwent thematic analysis.

**Results:**

Most community pharmacists perceived community pharmacy-based repeat medicine supply as beneficial, citing patient convenience in terms of accessibility (96.3%), time saving (95.6%) and reduced travel costs (88.5%). They viewed community pharmacies as suitable service points (94.3%) and supported appropriate remuneration for their services (90.6%). Fewer pharmacists considered potential barriers such as increased workload (44.8%), operating costs (39.8%), or space constraints (43.0%). Strongly endorsed facilitators included timely reimbursement to the pharmacists, clear procedures and guidelines, and structured collaboration with public facilities. A high proportion (88.4%) expressed willingness to participate. Qualitative findings reinforced perceived benefits for patients, community pharmacists, and public health system. Participants highlighted the importance of a clear operational framework, efficient reimbursement systems, and avoidance of previous pitfalls in public–private collaborations. While challenges were noted, pharmacists believed these could be mitigated through proper systems including communication platforms, scheduling mechanisms, and defined formularies.

**Discussion:**

Overall, community pharmacists demonstrated strong support for participating in repeat medicine supply services. Their motivation stems from patient benefits, professional fulfilment, and commercial sustainability. Realizing this model, however, requires system-level reforms, stakeholder engagement, and robust implementation mechanisms to ensure an effective and sustainable collaboration.

## 1 Introduction

Chronic diseases require long-term pharmacological treatment, often involving multiple medications taken continuously to maintain disease control and prevent complications. Ensuring consistent access to these medications is therefore essential for achieving optimal therapeutic outcomes and improving patients’ quality of life. As part of efforts to support medication adherence and the quality use of medicines, medications for chronic diseases are typically supplied on a monthly basis through public healthcare facilities in Malaysia such as hospitals and health clinics (1). Patients are subsequently scheduled for follow-up appointments to obtain their repeat medication supply.

This system supports regular medication monitoring and controlled dispensing. However, it may also present practical challenges for patients. Frequent visits to public healthcare facilities can be inconvenient, particularly for older persons, individuals with disabilities, and those facing transportation barriers. Moreover, this practice contributes to congestion, prolonged waiting times, and increased workload in outpatient pharmacy departments (1).

These challenges are especially evident in Malaysian public hospitals, which operate as integrated centers providing inpatient and outpatient services, follow-up care for discharged patients, medication dispensing, and repeat medication supply (2).

The increasing prevalence of non-communicable diseases (NCD) has further intensified the demand for services in public healthcare facilities. As more patients require long-term treatment and regular medication refills, healthcare systems face growing pressure to sustain efficient service delivery. In Malaysia, the number of patient visits to public healthcare facilities increased 4.5 times from 2008 to reach approximately 77 million visits in 2018, partly due to the rising burden of NCD requiring regular follow-up treatment and repeat medication supply (1). This trend has significantly contributed to congestion in hospitals and increased workload among healthcare personnel.

To address these operational challenges while maintaining continuity of medication supply, the Malaysian Ministry of Health (MOH) introduced several Value-Added Services (VAS) to complement conventional pharmacy counter dispensing. These services aim to provide patients with more convenient options to obtain their repeat medications. Examples include allowing patients to collect medicines from selected public healthcare facilities closer to their residence, drive-through counters, medication delivery via mail, and collection through designated lockers within hospitals (1).

The primary objective of VAS is to improve the efficiency of repeat medication supply while ensuring continuity of therapy and promoting medication adherence. These services are also expected to reduce waiting time, travel burden for patients, and congestion in public healthcare facilities (1).

However, some VAS models, such as drive-through counters, mail delivery, and locker collection systems, may limit direct interaction between patients and pharmacists (3–5). Such interaction is essential for effective medication counselling and ensuring safe medication use. Furthermore, switching between generic brands due to supply shortages may confuse patients when pharmacist consultation is unavailable (4). Even when medicines are correctly prescribed and dispensed, inadequate patient understanding of medication use may prevent therapeutic goals from being achieved (6). Chan et al. (2) also noted that many patients still prefer collecting medicines directly from hospital pharmacies to consult pharmacists. Other concerns include the inability to supply certain medications through VAS, particularly liquid formulations (2), medications requiring special storage conditions, and psychotropic drugs (7).

At the same time, community pharmacists in Malaysia remain relatively under-utilised within the healthcare system. Community pharmacies are commonly visited for purchasing medications, managing minor illnesses, or obtaining health supplements (8). However, community pharmacists possess the potential to play a greater role in chronic disease management and public health services (9). One major limitation is the absence of a clear separation between prescribing and dispensing practices in Malaysia, which limits opportunities for community pharmacists to provide pharmaceutical care services (10). Consequently, only a small proportion of prescriptions are filled at community pharmacies (8, 11, 12).

Public–private partnerships (PPP) have been increasingly recognised as an effective approach to improving healthcare delivery and expanding access to services. The World Bank defines PPP as a long-term partnership between the public and private sectors to deliver public services with shared risks and responsibilities (13). In healthcare systems where prescribing and dispensing are not formally separated, contracting community pharmacists to supply medications prescribed by public healthcare facilities represents a potential form of PPP. Such arrangements allow governments to leverage the accessibility, distribution networks, and professional expertise of community pharmacists to improve medication access and adherence while alleviating congestion in public healthcare facilities. Similar collaborations have been implemented internationally. For example, community pharmacies in Macau have been contracted to provide medication dispensing and pharmaceutical care services for patients from public healthcare facilities (14) while Jamaica’s National Health Fund collaborates with selected community pharmacies to provide additional prescription refill locations for patients (15). These models illustrate how public–private collaboration can expand access points for medication supply, a strategy that may be applicable to addressing congestion and improving service efficiency in Malaysia.

In Malaysia, PPP initiatives involving pharmacists have largely focused on health education programmes (16, 17) and smoking cessation services (18). Expanding PPP to involve community pharmacists in supplying repeat medications for patients with chronic diseases could potentially address gaps in the current system while reducing congestion in public healthcare facilities (4). Moreover, PPP has been recognized as an important mechanism supporting the Sustainable Development Goals (SDG), particularly SDG 17 which emphasizes partnerships between public and private sectors (19, 20). In Malaysia, outsourcing repeat medication supply from public healthcare facilities to community pharmacies has also been identified as an initiative under the Malaysian National Medicines Policy 2022–2026 to improve patient access to pharmacy services through PPP (21). However, despite this policy direction, implementation in Malaysia remains limited and not widely established, indicating a gap between policy intent and real-world practice.

Given the strategic location, professional training, and accessibility of community pharmacists, their involvement in such collaboration presents an opportunity to enhance medication access and pharmaceutical care. However, successful implementation requires careful consideration of the perspectives and readiness of community pharmacists.

This study aimed to examine community pharmacists’ perspectives on public–private collaboration in providing repeat medication supply for patients with chronic diseases in Malaysia.

## 2 Materials and methods

### 2.1 Design

This study employed a concurrent embedded mixed-methods design (22), with a quantitative-dominant (QUAN + qual) approach, in which the primary component was a cross-sectional quantitative survey and the secondary component comprised qualitative in-depth interviews embedded to provide explanatory insights. The quantitative study was designed to assess the perspectives of community pharmacists in Malaysia regarding their potential role in supplying repeat medicines to patients with chronic diseases from public health facilities, whereas the qualitative study explored their attitudes towards such a programme in greater depth. This mixed-methods approach was chosen because it allows for a more comprehensive understanding of the research problem than the use of either methods alone (22). The study was reported in accordance with the STROBE guideline for observational quantitative research (23) and the COREQ guideline for qualitative research (24).

### 2.2 Participants and sampling

The study was conducted among community pharmacists practising in Malaysia. For the quantitative component, participants were recruited from community pharmacies across all states in Malaysia using a nationwide online survey. The inclusion criteria were fully registered pharmacists working in community pharmacies in Malaysia, including those holding administrative responsibilities within their organisations. Pharmacy business owners who were not registered pharmacists and provisionally registered pharmacists were excluded.

A list of community pharmacists was obtained from an official Malaysian MOH, Pharmaceutical Services Programme dataset, downloaded and archived in PDF format on 29 March 2024 (Daftar Lesen Portal). This document contains the national listing of licensed community pharmacy premises and was used as the sampling frame for this study. A proportionate stratified sampling approach with quota control was used, whereby invitations were sent to pharmacists across all Malaysian states. Recruitment was monitored to ensure that responses reflected the proportional distribution of community pharmacists across states, and additional invitations were sent to under-represented strata until target proportions were achieved. For the qualitative component, participants were selected using maximum variation purposive sampling to capture a wide range of professional experiences and viewpoints. Pharmacists with variation in roles, practice settings and years of professional experience were invited to participate including those affiliated with relevant professional bodies such as the Malaysian Pharmacists Society (MPS), Malaysian Community Pharmacy Guild (MCPG), Sabah Pharmaceutical Society, and Sarawak Pharmaceutical Society. The same inclusion criteria was applied; fully registered pharmacists practising in community pharmacies in Malaysia, including those in administrative roles.

### 2.3 Sample size calculation

The sample size for the quantitative survey was calculated using the Raosoft® sample size calculator. A 95% confidence level and 5% margin of error were used, as these are widely accepted parameters in health research for balancing statistical precision and feasibility. Based on a study population of 4,796 community pharmacists in Malaysia as of 29 March 2024, a minimum required sample size was 356. For the qualitative component, sampling followed the principle of data saturation, whereby interviews were continued until no substantially new information or themes emerged from the data. Saturation was determined based on thematic repetition, whereby newly collected data repeatedly reflected existing codes and no new themes were generated (22).

### 2.4 Instruments used

For the quantitative study, a self-administered questionnaire in English was developed based on an extensive literature review, including Malaysian healthcare policies and guidance documents (1, 7, 21, 25, 26), studies on repeat medicines supply in public health facilities (2–4, 27–29), and literature on public–private collaboration (16, 20, 30–36). The Pharmacy Value-Added Services Questionnaire (PVASQ) was also used as a reference in questionnaire development (37). Following the literature review, questionnaire items were subsequently developed and refined through iterative discussions among the research team, ensuring alignment with the study objectives and relevance to community pharmacy practice in Malaysia. The final draft was prepared prior to expert content validation.

The draft questionnaire underwent content validation using the Content Validity Index (CVI) method. Feedback was obtained from eight experts and stakeholders, comprising five academicians experienced in community pharmacy research, two community pharmacists with more than 10 years of practice and experience in public–private collaboration, and one public hospital pharmacist with extensive research experience in repeat medicines supply. The average CVI was 0.975, while the universal agreement CVI was 0.80, indicating good content validity (38, 39).

A pilot test involving 10 community pharmacists was conducted to assess the feasibility of the study protocol and the applicability of the questionnaire. This sample size was considered appropriate for pilot testing aimed at instrument refinement, focusing on clarity, face validity, and feasibility prior to full-scale data collection. Cronbach’s alpha demonstrated good reliability for all constructs, including general perspectives (*α* = 0.890), barriers (*α* = 0.856), and facilitators (*α* = 0.839), indicating acceptable internal consistency across all domains. Data from the pilot study were not included in the main analysis. Following discussion within the research team, the questionnaire was revised and finalised. It comprised three sections: (i) perspectives, (ii) perceived benefits and facilitators, and (iii) demographic data. Responses on perspectives and perceived benefits were measured using a 5-point Likert-like scale ranging from Strongly Disagree (1) to Strongly Agree (5) (40). Perceived facilitators were rated on a 5-point scale from Not at All Important (1) to Very Important (5) (41). Willingness to participate in the collaboration was rated from Not Very Likely (1) to Very Likely (5) (42) (Supplementary File S1). Different Likert scale anchors were used across constructs to align with the conceptual nature of each domain, with agreement scales used for attitudinal items and importance scales used for perceived facilitators.

For the qualitative study, an interview topic guide was developed from the literature review. The guide was reviewed by two experts in pharmacy research guided by the Interview Protocol Refinement Framework (43), which supports systematic expert review of interview protocols to ensure alignment between the interview questions and the research objectives. Probes were included where necessary to support richer and more detailed discussion (Supplementary File S2).

### 2.5 Data collection

For the quantitative study, the online questionnaire was hosted on SurveyMonkey®. The survey weblink was sent to eligible community pharmacists using the retrieved contact list. To reduce the likelihood of duplicate responses, the “multiple responses” feature in SurveyMonkey® was disabled, allowing only one response per browser or email address.

For the qualitative study, selected participants were invited for one-to-one in-depth interviews. Interviews were conducted either face-to-face or online via Zoom, depending on participants’ availability and preference. Participants were reminded to share their views based on their professional experience and were assured of anonymity throughout the study and reporting. All interviews were conducted in English, audio-recorded with participant permission, and transcribed verbatim. The transcripts were subsequently checked by a research assistant to ensure transcription accuracy.

Interviews were conducted by PL, a retired community pharmacist with over 20 years of professional experience and prior training in qualitative research. Some participants were known to the interviewer in a professional capacity. Reflexivity was maintained through the use of a structured interview guide, regular discussions within the research team during analysis, and maintenance of a reflexive journal and audit trail.

### 2.6 Data analysis

Quantitative data from the online questionnaire were analysed using the Statistical Package for the Social Sciences (SPSS) version 29.0 (IBM Corp., Armonk, New York). Descriptive statistics were used for all variables. For numerical data, means and standard deviations were calculated. Inferential analysis included the Chi-square test and binary logistic regression to examine associations between variables. Binary logistic regression analyses were conducted based on the study objectives, with separate models examining (i) the association between demographic characteristics and willingness to participate, and (ii) the association between perspectives and barriers with willingness, with variables entered simultaneously using the enter method. A *p*-value of less than 0.05 was considered statistically significant. Responses to open-ended survey questions were analysed separately using thematic analysis as supplementary qualitative data and were not integrated with the qualitative interview dataset.

For the qualitative component, interview transcripts were analysed using the thematic analysis approach of Braun and Clarke (44, 45). The analysis involved six phases: data familiarisation, generation of initial codes, searching for themes, reviewing themes, defining and naming themes, and producing the report. Familiarisation began with repeated listening to the audio recordings and careful reading of the transcripts. QDA Miner Lite (46) was used to support the initial coding of textual data, after which theme development and review were undertaken by the research team. The study applied thematic analysis to identify patterns in the data, with interpretation of participants’ perspectives in relation to healthcare practice and service implementation (44).

### 2.7 Ethical consideration

Ethical approval for this study was obtained from the Human Ethics Committee of Taylor’s University (HEC 2023/299). Informed consent was obtained digitally for the quantitative survey participants, while respondent anonymity was preserved through the “anonymous responses collector” function in SurveyMonkey®. For the qualitative component, participants provided informed consent prior to the interview, and their anonymity was protected by assigning each participant a code number (47). Permission to audio-record the interview was obtained prior to each interview. All study data were stored in password-protected files on a secure, access-restricted institutional drive. Only the research team had access to the data, and all identifiable information was removed during transcription to ensure confidentiality. Consent forms were stored separately from research data.

## 3 Results

### 3.1 Quantitative results

#### 3.1.1 Demographic characteristics of participants

**Figure 1:**
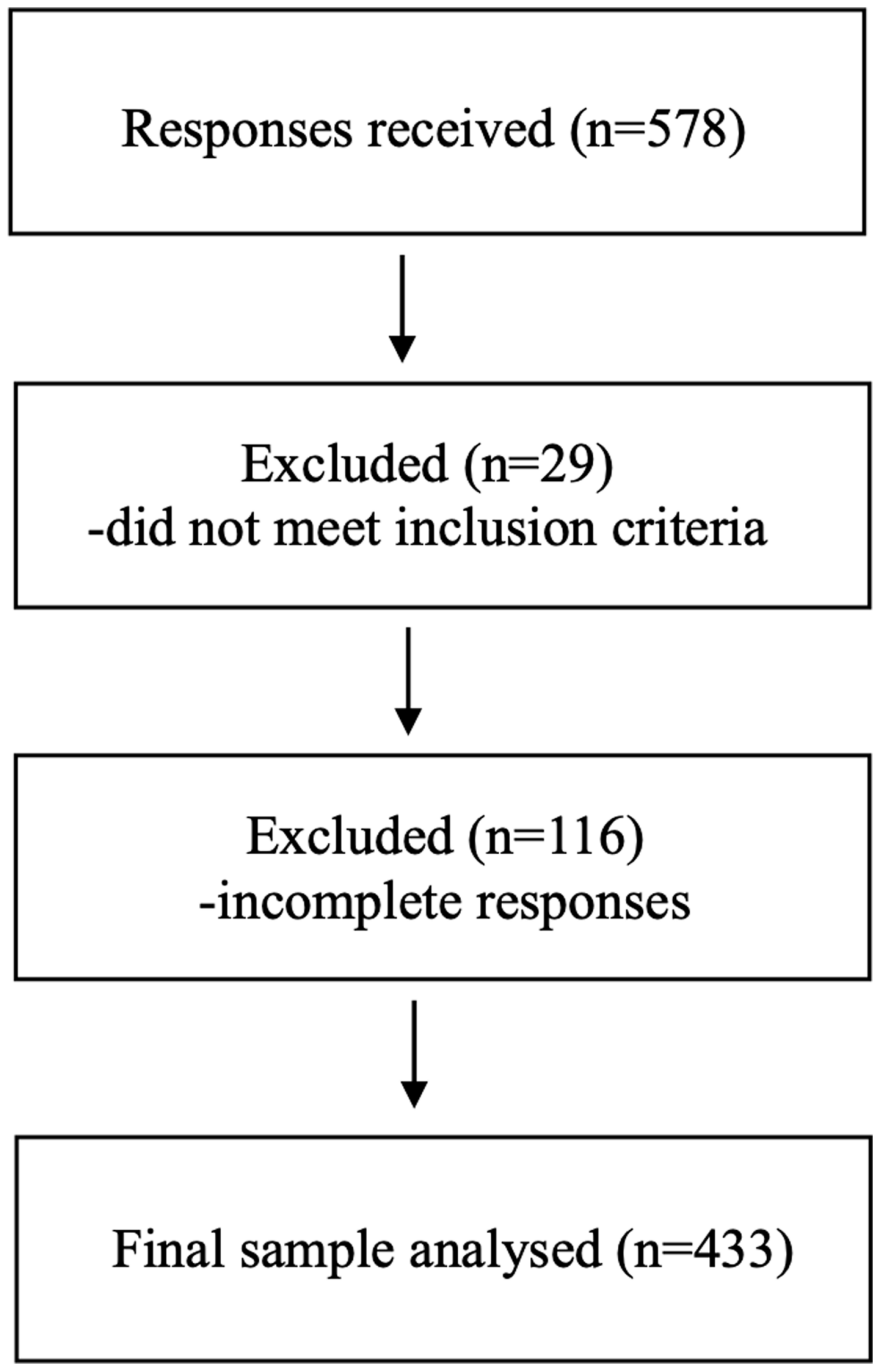
Flow diagram of survey responses.

The weblink to the online questionnaire was distributed to 2,506 community pharmacists between January and June 2024. A total of 578 responses were received. Of these, 29 respondents were excluded because they did not meet the study’s inclusion criteria, and 116 responses were removed due to incomplete data, resulting in 433 completed questionnaires being included in the final analysis (Figure 1). This corresponds to a response rate of 17.3%.

The demographic characteristics of the respondents are presented in Table 1. The participants had a mean age of 41.53 years (SD = 10.96; range: 25–77 years). The average duration of practice in community pharmacy was 13.43 years (SD = 9.87; range: 1–50 years).

**Table 1:** Characteristics of the participants (*N* = 433).

Characteristics	Number of respondents (%)
Gender	
Female	276 (63.7%)
Male	157 (36.3%)
Age group (years)	
21–29	48 (11.1%)
30–39	165 (38.1%)
40–49	108 (24.9%)
50–59	80 (18.5%)
60 or older	32 (7.4%)
Ethnic group	
Malay	113 (26.1%)
Chinese	299 (69.1%)
Indian	14 (3.2%)
Others^a^	7 (1.6%)
Country of pharmacy degree	
Malaysia	300 (69.3%)
Overseas	133 (30.7%)
Highest education level	
Basic pharmacy degree	396 (91.5%)
Postgraduate Master degree^b^	37 (8.5%)
Years of working in community pharmacy in Malaysia	
< 5	103 (23.8%)
5 to 9	89 (20.5%)
10 to 14	66 (15.2%)
15 to 19	32 (7.4%)
20 to 24	68 (15.7%)
25 to 29	44 (10.2%)
≥ 30	31 (7.2%)
Type of employment	
Full-time employed pharmacist	238 (55.0%)
Employer or shareholder pharmacists	195 (45.0%)
Job position	
Manager	367 (84.8%)
Non-manager	66 (15.2%)
Job scope^c^	
Retail at outlet	413 (95.4%)
Wholesales at outlet or headquarters	61 (14.1%)
Purchasing at headquarters	78 (18.0%)
Marketing at headquarters	44 (10.2%)
Administration at headquarters	79 (18.2%)
Workplace location	
Selangor	108 (24.9%)
Sarawak	54 (12.5%)
Kuala Lumpur	46 (10.6%)
Johor	36 (8.3%)
Sabah	30 (6.9%)
Penang	28 (6.5%)
Perak	24 (5.5%)
Kedah	21 (4.9%)
Kelantan	19 (4.4%)
Pahang	17 (3.9%)
Negeri Sembilan	15 (3.5%)
Melaka	14 (3.2%)
Terengganu	14 (3.2%)
Perlis	3 (0.7%)
Labuan	2 (0.5%)
Putrajaya	2 (0.5%)

a Others includes Bumiputra Sabah, Dusun, Melanau, Sino-Kadazan and undisclosed.

b Includes pharmacy, health and non-health related postgraduate master degrees.

c Multiple responses allowed for each respondent.

#### 3.1.2 General perspectives

**Figure 2:**
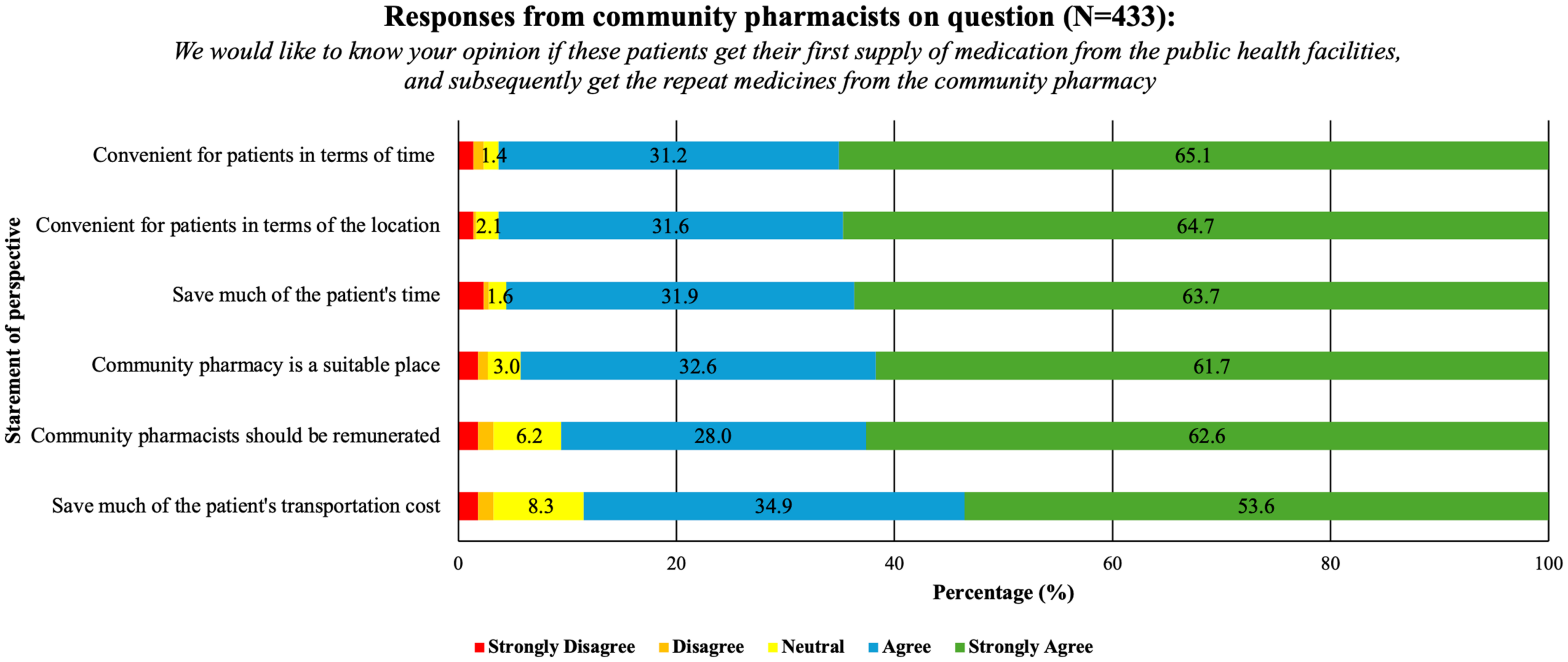
Community pharmacists’ perspectives on providing repeat medicines supply (*N* = 433).

Overall, 88.5–96.3% of community pharmacists responded either agree or strongly agree across the six perspective statements regarding the provision of repeat medicines supply to patients from public health facilities (Figure 2), indicating generally favourable responses.

The statements related to patient convenience received the highest mean scores. In particular, pharmacists agreed that obtaining repeat medicines from community pharmacies would be convenient for patients in terms of operating hours (mean = 4.58, SD = 0.70; 96.3% agreement) and convenient in terms of location when patients are allowed to choose their preferred community pharmacy (mean = 4.58, SD = 0.68; 96.3% agreement). Similarly, respondents expressed agreement that the service could save patients’ time (mean = 4.54, SD = 0.77; 95.6% agreement) and that community pharmacies are suitable locations for providing repeat medicines supply services (mean = 4.51, SD = 0.77; 94.3% agreement). Notably, pharmacists generally agreed that community pharmacists should be remunerated for providing repeat medicines supply services (mean = 4.48, SD = 0.83; 90.6% agreement), indicating support for an appropriate compensation mechanism if such a public–private collaboration were to be implemented.

#### 3.1.3 Perceived barriers

**Figure 3:**
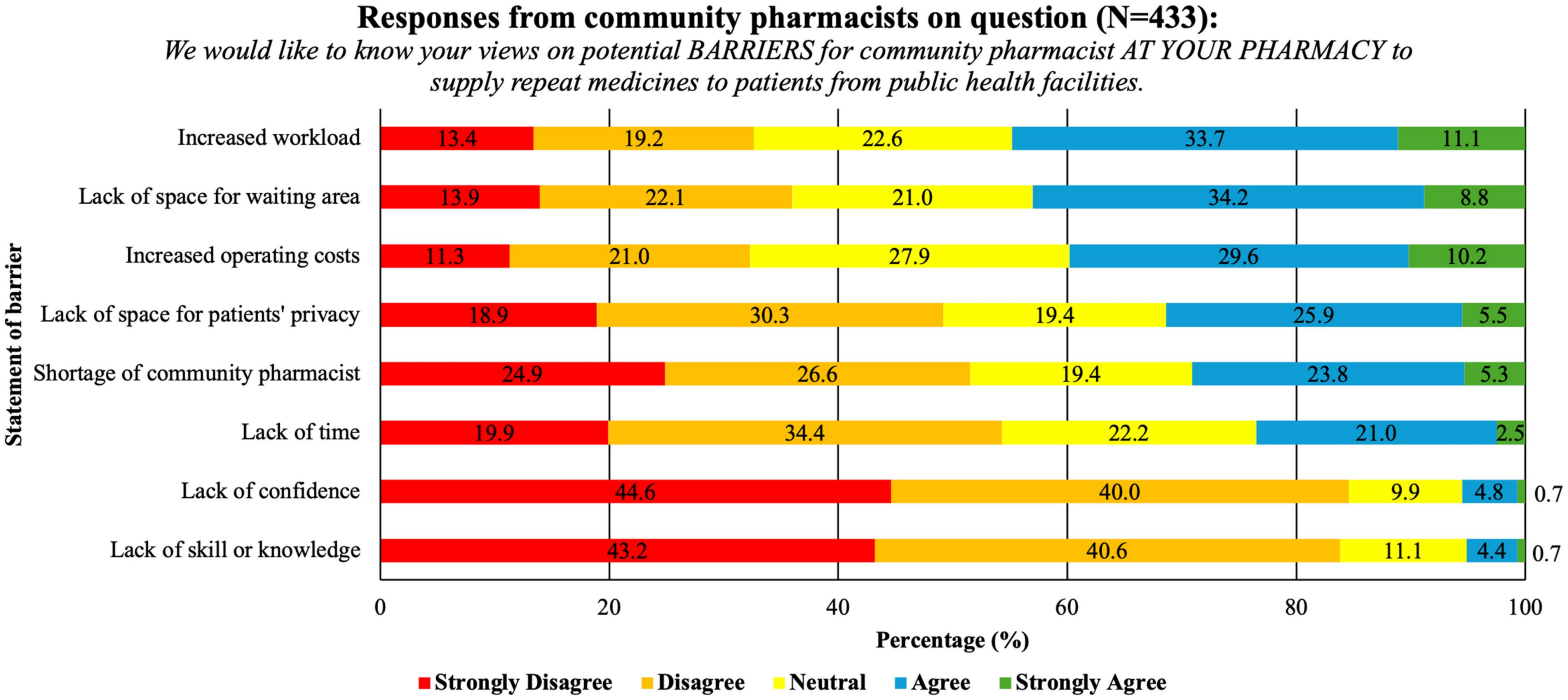
Community pharmacists’ perceived barriers on providing repeat medicines supply (*N* = 433).

In general, fewer than half of the community pharmacists agreed with any of the eight proposed barrier statements (Figure 3). Among the barriers examined, increased workload for community pharmacists recorded the highest mean score (mean = 3.10, SD = 1.23), with 44.8% of respondents indicating agreement. This was followed by lack of space for a waiting area (mean = 3.02, SD = 1.21; 43.0% agreement) and increased operating costs (mean = 3.06, SD = 1.17; 39.8% agreement).

Participants were also invited to report additional barriers not listed in the questionnaire. A small proportion of pharmacists (33 respondents; 7.6%) highlighted stock-related issues, including difficulty in identifying medication source, limited stock availability, limited storage capacity at the pharmacy and management of uncollected medications. In addition, 16 pharmacists (3.7%) identified patients’ lack of awareness and expectations as a potential barrier. These concerns included patients’ understanding of prescription validity, reluctance to pay for services, expectations for immediate service, and uncertainty about which pharmacy to visit for repeat medicine collection. Responses to open-ended survey questions were analysed separately as a secondary qualitative component and were not integrated with the primary qualitative interview dataset.

#### 3.1.4 Perceived facilitators

**Figure 4:**
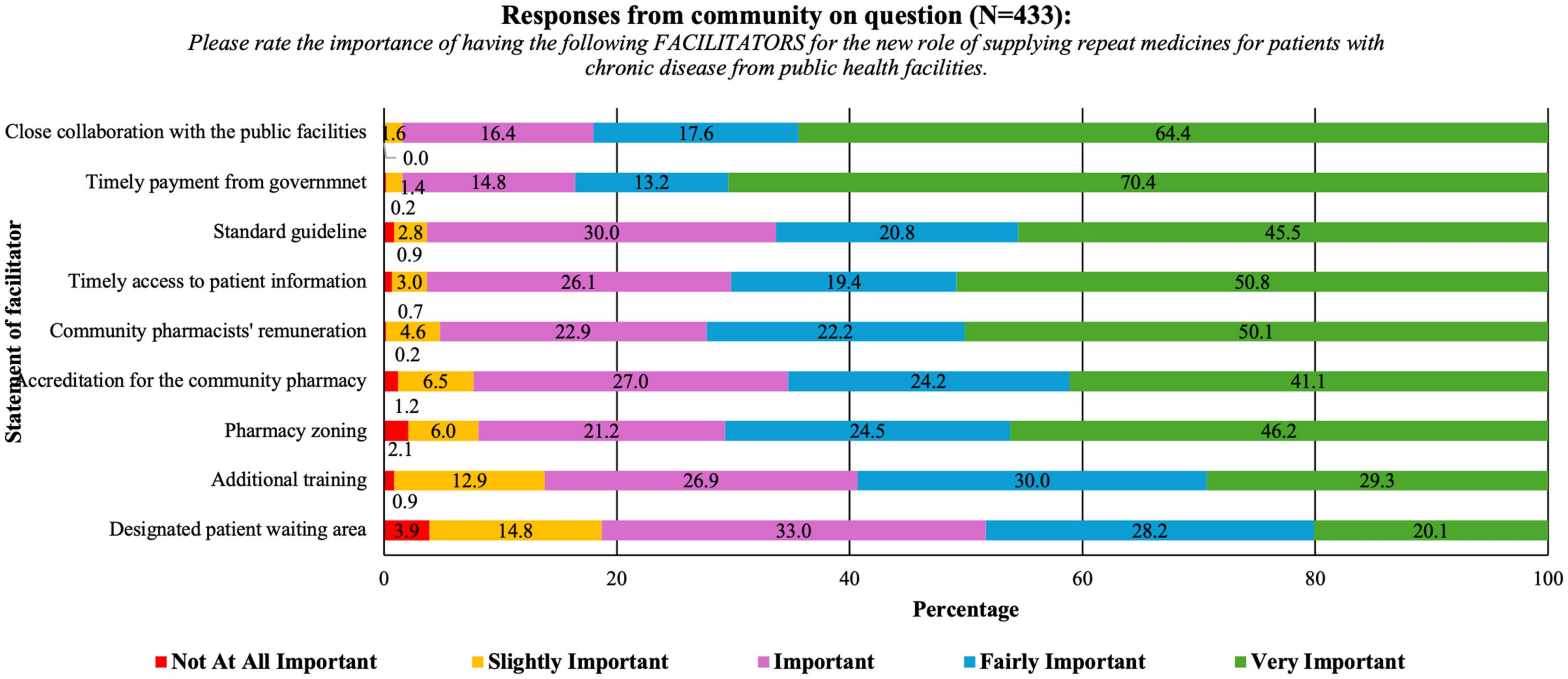
Community pharmacists’ perceived facilitators on providing repeat medicines supply (*N* = 433).

Collectively, more than 80% of community pharmacists rated all nine proposed facilitators as Important, Fairly Important, or Very Important (Figure 4). Based on mean scores, timely payment from the government to community pharmacies, particularly where reimbursement of medicine costs is involved, was the highest-rated facilitator (mean = 4.52, SD = 0.81). Close collaboration with public health facilities in addressing patients’ medication-related issues was also highly rated (mean = 4.45, SD = 0.82). Other facilitators with relatively high mean scores included timely access to patient information through electronic medical records (mean = 4.17, SD = 0.96), appropriate remuneration for community pharmacists providing the service (mean = 4.17, SD = 0.95), and the availability of standardised guidelines outlining the required services (mean = 4.07, SD = 0.97).

In addition, respondents were invited to suggest other facilitators through an open-ended question. Additional suggestions provided included the need for amendments to pharmacy laws to facilitate programme implementation, strong government commitment, indemnity coverage for community pharmacists, and public awareness initiatives to improve understanding and acceptance of the service. Open-ended responses related to facilitators were analysed separately as a secondary qualitative component and were not integrated with the primary qualitative interview dataset.

#### 3.1.5 Pharmacist’s remuneration and willingness to participate

**Figure 5:**
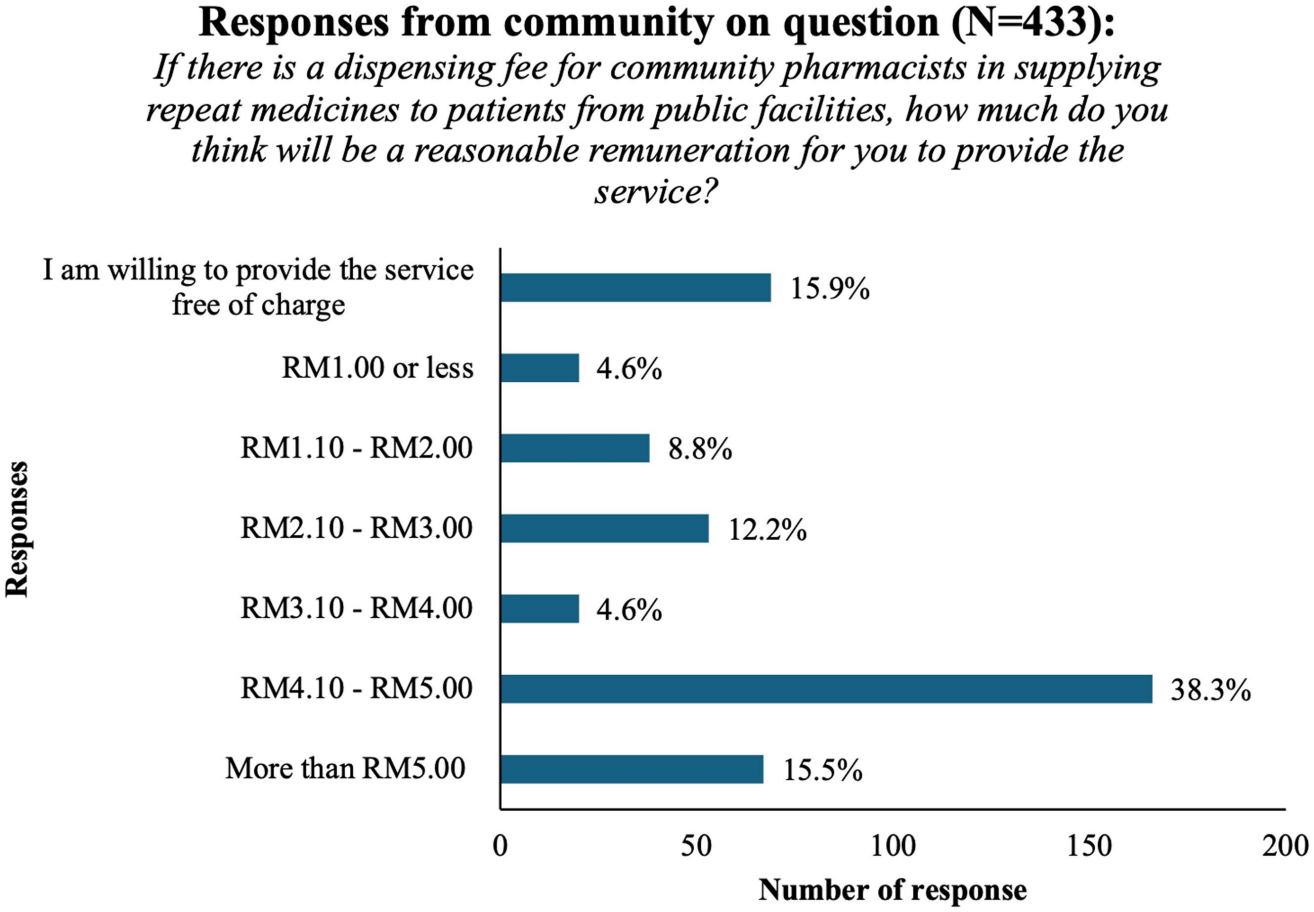
Community pharmacists’ responses on remuneration per occasion (*N* = 433).

Most community pharmacists (*n* = 166; 38.3%) indicated that a reasonable remuneration for supplying repeat medicines would range between RM4.10 and RM5.00 per occasion (Figure 5). A smaller proportion of respondents (*n* = 69; 15.9%) reported that they would be willing to provide the service without remuneration.

For the purpose of binary logistic regression analysis, the Likert-scale responses for willingness of community pharmacists to participate in the proposed public–private collaboration were recoded into a binary variable, with “Yes” (Very Likely and Somewhat Likely) and “No” (Neutral, Somewhat Unlikely, and Not Very Likely) to facilitate analysis. Based on this categorization, 383 pharmacists (88.4%) reported “Yes” to participating in supplying repeat medicines to patients with chronic diseases from public health facilities (mean = 4.40, SD = 0.86).

##### 3.1.5.1 Association of demographic variables with the willingness

The association between pharmacists’ demographic characteristics and their willingness to participate was examined using the Chi-square test. The inferential analysis showed that none of the demographic variables were significantly associated with willingness to participate, as all p values exceeded 0.05 (Table 2). This indicates that pharmacists’ willingness to engage in the collaboration was not influenced by their demographic characteristics.

**Table 2:** Association of demographic variables with willingness to participate.

Predictive factors	Rank	Frequency of willingness (%)		*p*-value
		Yes	No	
Gender	Female	246 (89.1%)	30 (10.9%)	0.558
	Male	137 (87.3%)	20 (12.7%)	
Age group	21–29	44 (91.7%)	4 (8.3%)	0.517
	30–39	141 (85.5%)	24 (14.5%)	
	40–49	99 (91.7%)	9 (8.3%)	
	50–59	70 (87.5%)	10 (12.5%)	
	60 and above	29 (90.6%)	3 (9.4%)	
Ethnic	Malay	103 (91.2%)	10 (8.8%)	0.678
	Chinese	261 (87.3%)	38 (12.7%)	
	Indian	13 (92.9%)	1 (7.1%)	
	Others^a^	6 (85.7%)	1 (14.3%)	
Country of pharmacy degree	Local	265 (88.3%)	35 (11.7%)	0.907
	Overseas	118 (88.7%)	15 (11.3%)	
Highest education level	Basic pharmacy degree	349 (88.1%)	47 (11.9%)	0.494
	Postgraduate master degree	34 (91.9%)	3 (8.1%)	
Years of working in community pharmacy in Malaysia	< 5	92 (89.3%)	11 (10.7%)	0.519
	5 to 9	75 (84.3%)	14 (15.7%)	
	10 to 14	59 (89.4%)	7 (10.6%)	
	15 to 19	31 (96.9%)	1 (3.1%)	
	20 to 24	62 (91.2%)	6 (8.8%)	
	25 to 29	38 (86.4%)	6 (13.6%)	
	≥ 30	26 (83.9%)	5 (16.1%)	
Type of employment	Full-time employed pharmacist	209 (87.8%)	29 (12.2%)	0.647
	Employer or shareholder pharmacists	174 (89.2%)	21 (10.8%)	
Job position	Manager	327 (89.1%)	40 (10.9%)	0.320
	Non-manager	56 (84.8%)	10 (15.2%)	
Workplace region^b^	Northen	64 (84.2%)	12 (15.8%)	0.316
	Central	155 (90.6%)	16 (9.4%)	
	East coast	47 (94.0%)	3 (6.0%)	
	Southern	44 (88.0%)	6 (12.0%)	
	Eastern Malaysia	73 (84.9%)	13 (15.1%)	

a Others includes Bumiputra Sabah, Dusun, Melanau, Sino-Kadazan and undisclosed.

b Workplace location is grouped by regions of Northen (Kedah, Perlis, Penang, Perak), East coast (Kelantan, Pahang, Terengganu), Southern (Johor, Melaka) and Eastern Malaysia (Labuan, Sabah, Sarawak).

##### 3.1.5.2 Association between perspectives, barriers and the willingness

Factor analysis was conducted on the community pharmacists’ responses to statements on perspectives and perceived barriers using principal component analysis (PCA) with Promax rotation. Sampling adequacy was assessed using the Kaiser–Meyer–Olkin (KMO) measure, and Bartlett’s test of sphericity was performed to confirm suitability for factor analysis. Factors were retained based on eigenvalues greater than 1.0. Factor loadings of ≥ 0.30 were considered significant, and coefficients below this threshold were suppressed to aid interpretation.

The six perspective statements were grouped into a single underlying domain, indicating that they collectively represented a unified construct reflecting pharmacists’ overall perspectives towards providing repeat medicines supply. In contrast, the eight barrier statements were grouped into two distinct domains: perceived system barriers and personal barriers. The personal barriers domain comprised statements related to lack of skill or knowledge and lack of confidence among community pharmacists in providing the service.

For further analysis, willingness to participate in the public–private collaboration was categorized as “Yes” if respondents selected somewhat Likely or Very Likely, and “No” otherwise. The scores for perspectives and barriers were computed as summated scores. Binary logistic regression analysis was then performed to examine the association between these variables and pharmacists’ willingness to participate.

Among the variables analysed, perspectives and perceived system barriers were found to be significantly associated with willingness to participate (Table 3). Higher perspective scores were associated with increased odds of willingness (OR = 2.234, 95% CI: 1.493–3.343, *p* < 0.001), while higher perceived system barriers were associated with decreased odds of willingness (OR = 0.505, 95% CI: 0.337–0.757, *p* = 0.001). Perceived personal barriers were not significantly associated with willingness (OR = 0.895, 95% CI: 0.609–1.315, *p* = 0.572). The model demonstrated an adequate goodness-of-fit, as indicated by the Hosmer–Lemeshow test (*p* = 0.443), which exceeded the threshold of 0.05. The Nagelkerke *R^2^* value of 0.163 suggested that the model explained a modest proportion of the variance in willingness to participate.

**Table 3:** Association between perspectives, barriers and the willingness to participate.

Predictors	B	SE	Wald	*p*-value	Odd ratio (95% CI)	Hosmer and lemeshow goodness-of-fit test	
						Chi-square	*p*-value
Perspectives	0.804	0.206	15.259	0.000	2.234 (1.493, 3.343)	7.905	0.433
System barriers	−0.682	0.206	10.949	0.001	0.505 (0.337, 0.757)		
Personal barriers	−0.111	0.196	0.319	0.572	0.895 (0.609, 1.315)		

Binary logistic regression analysis identified perspectives and perceived system barriers as significant predictors of willingness to participate in supplying repeat medicines from public health facilities. The model demonstrated good fit as indicated by the Hosmer–Lemeshow test (*χ^2^* = 7.905, *p* = 0.433).

### 3.2 Qualitative results

#### 3.2.1 Demographic characteristics of interviewed participants

Between December 2023 and May 2024, a total of 15 one-to-one in-depth interviews, each lasting 25–60 min, were conducted. Data collection ceased once data saturation was achieved. Among these interviews, three were conducted face-to-face, while the remaining 12 were conducted online via the Zoom platform. The participating pharmacists were aged 29 to 69 years, with community pharmacy practice experience in Malaysia ranging from 3 to 41 years (Table 4).

**Table 4:** Characteristics of the interviewed participants (*N* = 15).

Characteristics	Number of respondent (%)
Age (years)	
Below 30	1 (6.7%)
30 to 39	1 (6.7%)
40 to 49	5 (33.3%)
50 to 59	5 (33.3%)
60 and above	3 (20.0%)
Gender	
Female	8 (53.3%)
Male	7 (46.7%)
Highest pharmacy education	
Bachelor degree	14 (93.3%)
Postgraduate	1 (6.7%)
Main pharmacy job scope	
Retail	7 (46.7%)
Retail and wholesales	5 (33.3%)
HQ administration	3 (20.0%)
Job position	
Managerial role	14 (93.3%)
Non-managerial role	1 (6.7%)
Type of employment	
Self-employed/Shareholder	11 (73.3%)
Full time employee	4 (26.7%)
Pharmacy ownership	
Individual pharmacist	7 (46.7%)
Group of pharmacists	2 (13.3%)
Corporate bodies	6 (40.0%)
Years of practice in community pharmacy in Malaysia	
Less than 10	1 (6.7%)
10 to 19	4 (26.7%)
20 to 29	7 (46.7%)
30 to 39	2 (13.3%)
40 and above	1 (6.7%)

Three core categories of themes emerged from the analysis: (1) Perceived benefits, (2) Implementation aspects to be considered and (3) Community pharmacists’ motivation to participate. A summary of the findings is presented in Table 5.

**Table 5:** Themes and subthemes identified from thematic analysis.

**Themes**	**Subthemes**
Perceived benefits	Improve patient's access to better care
	Improve patient's convenience
	Enhance community pharmacist's professional role and image
	Increase community pharmacy commercial success
	Reduce healthcare burden in public sector
	Positive impact on public health facilities manpower
Implementation aspects to be considered	Standard operation procedure and requirements for service provision
	Patient appointment system
	Participating pharmacy database
	Platform for communication
	Medication supply
	Drug costs and professional fees
Community pharmacists' motivation to participate	-

#### 3.2.2 Theme 1: perceived benefits

##### 3.2.2.1 Improved patient’s access to better care

Community pharmacies are conveniently located within neighborhoods and are generally less crowded than public health facilities, making them more accessible for patients seeking pharmacy services. Unlike public facilities, where patients may encounter different pharmacists due to shift rotations, community pharmacies often provide continuity of care through the same pharmacist, enabling stronger pharmacist–patient relationships. This setting allows pharmacists to spend more time with patients and encourages open discussion about health concerns.

*“Hospital pharmacists work in shifts, limiting continuity of care. In community pharmacies, patients usually see the same pharmacist, which improves continuity.”* [CP07; 56 year-old, 28 years of experience].

Pharmacists also viewed their role in repeat medicine supply as extending beyond medication dispensing to comprehensive pharmaceutical care, including medication review, counselling, and promoting adherence and safety. They also highlighted their contribution to chronic disease management by supporting patient self-care through routine monitoring services commonly available in community pharmacies, such as blood glucose testing, blood pressure measurement, and weight monitoring.

*“We provide counselling and basic monitoring services such as blood pressure, body weight, and blood glucose testing, which can indirectly improve disease outcomes.”* [CP06; 41 year-old, 12 years of experience].

##### 3.2.2.2 Improve patient’s convenience

Community pharmacists strongly believed that allowing patients to refill repeat medicines at community pharmacies would significantly improve patient convenience. This improvement relates mainly to reduced travel time and costs, greater time efficiency, and better alignment with patients’ work schedules. This convenience was consistently linked to easier access through neighbourhood-based services and extended operating hours, which better align with patients’ daily routines.

This was reflected in pharmacists’ accounts highlighting reduced travel time and expenses when medicines are obtained closer to home or workplace:

*“And then for the patients, because they only need to go to their neighbourhood pharmacies to collect the medicines, so most likely will be nearer to the place they stay, they can cut down in the travelling time and expenses.”* [CP01; 55 year-old, 25 years of experience].

Beyond geographical accessibility, pharmacists also emphasised temporal convenience, particularly for working adults and caregivers who benefit from extended pharmacy operating hours:

*“This will benefit the rakyat (citizen in Malay) the most, not just for the patients but also for their caregivers to collect the medicines at community pharmacy, especially out of working hours, because community pharmacies, they open at night as well, until 9:00 pm.”* [CP06; 41 year-old, 12 years of experience].

Collectively, these views demonstrate that improved convenience is not limited to physical proximity, but also includes temporal flexibility, making community pharmacies a more accessible option compared to traditional public health facilities.

##### 3.2.2.3 Enhance community pharmacist’s professional role and image

Community pharmacies are well positioned to serve public healthcare needs; however, the relatively low number of prescriptions filled in community pharmacies limits pharmacists’ opportunities to contribute more broadly as recognised healthcare professionals. Increased patient visits, particularly from public health facilities, would enable pharmacists to expand their services to the community, including providing education on non-communicable diseases (NCD) and promoting healthy lifestyles. Pharmacists also believed that greater interaction with patients would enhance public awareness of the professional roles of community pharmacists—an area they felt remains under-recognised. While pharmacists expressed confidence in their training and experience in dispensing medicines and providing medication counselling, many perceived that their professional capabilities are currently underutilised within the existing practice environment.

*“It provides an opportunity to promote NCD awareness and enhances our professional role, leading to greater job satisfaction, especially among younger pharmacists.”* [CP08; 69 year-old, 41 years of experience].

*“We are as qualified as pharmacists in public facilities but remain underutilised, with limited opportunities to serve the public. Expanding the professional role of community pharmacists is therefore necessary.”* [CP08; 69 year-old, 41 years of experience].

##### 3.2.2.4 Increase community pharmacy commercial success

Community pharmacists acknowledged that collaboration with the government could also bring commercial benefits by increasing customer traffic to their pharmacies. Greater patient visits may create opportunities to recommend appropriate health-related products that support patients’ wellbeing while contributing to pharmacy revenue. In addition, pharmacists noted that managing a higher volume of patients with chronic diseases may require employing more pharmacists, potentially creating additional employment opportunities within the community pharmacy sector.

*“Besides getting the repeat prescriptions, we also get new customers from surrounding areas. From there we can enhance our sales by recommending the supplements or others to them to improve their health. So, I think it’s good for community pharmacy.”* [CP02; 54 year-old, 25 years of experience].

*“This will create more job opportunities because more pharmacists are required to be based at the community pharmacies to serve the patients from the government hospitals.”* [CP06; 41 year-old, 12 years of experience].

##### 3.2.2.5 Reduce healthcare burden in public sector

Community pharmacists believed that involving them in supplying repeat medicines could help decongest public health facilities and reduce the heavy workload faced by pharmacists in the public sector. They also highlighted that greater engagement of community pharmacists in patient care could contribute to improved medication adherence and disease management, which in turn may reduce hospital readmissions and medicine wastage, thereby supporting national healthcare outcomes.

*“Well, I think it’s a good move because the government hospital is a bit congested and patients need to wait for a long time. So if the government outsource this service to the community pharmacists, this should reduce the staff burden and the hospital congestion.”* [CP03; 46 year-old, 21 years of experience].

*“With better disease outcomes from community pharmacy cares, we should reduce the patient’s hospital readmission. Community pharmacists will also help to reduce the healthcare cost from poor medication compliance that leads to medicines expiry and wastage, besides readmission”* [CP06; 41 year-old, 12 years of experience].

##### 3.2.2.6 Positive impact on public health facilities manpower

Many community pharmacists believed that involving community pharmacies in repeat medicines supply could reduce the workload of healthcare personnel in public health facilities. When asked about the potential impact on pharmacist employment in the public sector, opinions were mixed. Some participants felt that such collaboration might reduce manpower needs and operational costs in public facilities. However, others disagreed, emphasizing that hospital pharmacists remain essential for clinical pharmacy services, particularly for inpatient care. They suggested that reducing responsibilities related to repeat medicines supply could instead allow hospital pharmacists to devote more time to patient counselling and clinical services. Overall, respondents viewed the potential manpower implications as beneficial to the public healthcare system.

*“I think it definitely will reduce the manpower needed at the hospital pharmacy department. If you have less work, then you don’t need so many people to work in the department. This is a fact.”* [CP09; 60 year-old, 33 years of experience].

*“Dispensing is only one aspect of a pharmacist’s role. Shifting repeat refills to community pharmacies would allow hospital pharmacists to focus on higher-value care and is unlikely to reduce headcount; it may even expand their roles.”* [CP05; 35 year-old, 14 years of experience].

#### 3.2.3 Theme 2: implementation aspects to be considered

##### 3.2.3.1 Standard operation procedure and requirements for service provision

Community pharmacists emphasized the need for a government-issued standard operating procedure (SOP) to guide their participation in repeat medicines supply. Such guidelines should clearly outline the process flow for medicine supply, the roles and responsibilities of pharmacists and patients, and the scope of pharmacy services expected. Pharmacists also noted that a well-defined SOP would help determine appropriate pharmacy infrastructure, staffing requirements, and the need for professional indemnity coverage.

Collectively, community pharmacists’ views extend beyond procedural guidance to broader policy and regulatory considerations. These include the need for clear accountability in medication-related errors, as well as operational requirements such as patient flow management and service infrastructure within community pharmacies. This indicates that policy development should not be limited to clinical processes alone, but should also address governance, safety, and service delivery design to ensure safe and practical implementation.

*“Clear policies and guidelines are needed to avoid ambiguity in medication errors, particularly regarding whether responsibility lies with the prescriber, dispensing pharmacist, or patient.”* [CP05; 35 year-old, 14 years of experience].

*“Government has to come up with the SOP for us to decide if we need certain areas for customers to queue, like having chairs, a waiting area or what. Otherwise, together with our existing customers, it will be a bit crowded. So we have to think about it.”* [CP02; 54 year-old, 25 years of experience].

##### 3.2.3.2 Patient appointment system

Community pharmacists highlighted the importance of establishing an integrated patient appointment system linked to a medicines database. Access to information on scheduled patient visits and required repeat medications would enable pharmacies to better prepare for service delivery. Such a system would support efficient stock management, ensuring that necessary medicines are available when patients arrive, and help ensure that pharmacists are available on duty to attend to patients at the scheduled time.

*“Access to limited patient data would help pharmacists anticipate refills, prevent stock shortages, and support medication adherence.”* [CP01; 55 year-old, 25 years of experience].

##### 3.2.3.3 Participating pharmacy database

Community pharmacists highlighted the need for a clear referral mechanism to guide patients from public health facilities to participating community pharmacies. They suggested that public health facilities provide a list of participating pharmacies, allowing patients to choose their preferred pharmacy without undue influence from any party and ensuring medicines are consistently collected from the selected location to facilitate stock management. Although pharmacists acknowledged that community pharmacies are less available in suburban and rural areas, they did not consider this a major barrier. Instead, they proposed that the programme could initially be implemented in urban areas with higher pharmacy density, while maintaining existing repeat medicine supply practices in rural settings through a hybrid implementation approach.

*“If the patient has already gotten the medicines from one pharmacy, make sure they will go to that pharmacy every time. We have stocked up certain medicine but if the patient doesn’t come back to our pharmacy and went to others. Then we will be stuck with the medicines, right?”* [CP02; 54 year-old, 25 years of experience].

*“In rural areas without community pharmacies, patients collecting repeat medicines from public health facilities remains acceptable, making a hybrid model more appropriate in the current setting.”* [CP05; 35 year-old, 14 years of experience].

##### 3.2.3.4 Platform for communication

Community pharmacists emphasised the need for an effective communication platform linking public health facilities, community pharmacies, and prescribers to ensure the smooth implementation of the repeat medicines supply programme. Strong communication would facilitate interprofessional collaboration, enabling community pharmacists to update healthcare providers on patients’ conditions that may require medical attention and to clarify any medication-related issues in prescriptions.

*“If patient’s follow-up with the hospital is in June but we see the patients in April noticing changes in their conditions, and then without having a communication platform between the community pharmacy and the hospital, it’s quite hard for us to provide updates on the patients. We need to send a message to the government to follow up or to report the unexpected changes in the patients, right?”* [CP12; 29 year-old, 3 years of experience].

*“A system that is shared among the government and private sectors would be the best. If we can access the patients’ EMR (electronic medical records), this will reduce not only the unnecessary calls to the prescribers but time spent just contacting them.”* [CP06; 41 year-old, 12 years of experience].

##### 3.2.3.5 Medication supply

Community pharmacists expressed uncertainty about how repeat medicine stocks should be sourced under the programme, particularly whether medicines would be supplied by public health facilities or purchased directly from pharmaceutical suppliers. Many suggested that obtaining medicines through government hospitals could be beneficial, as the government typically secures lower prices through bulk procurement and tendering processes. To prevent higher reimbursement costs, pharmacists emphasised the importance of accessing medicines at the same tendered prices as public health facilities, possibly through government-supported negotiations or professional body involvement. Alternatively, they proposed a pooled procurement model, where the government purchases medicines centrally and supplies them to participating community pharmacies.

*“To order the repeat medicines directly from suppliers, the pharmacies need to have a strong cash flow to purchase in bulk for lower medicine cost, and still may not get the contract price the hospital is getting.”* [CP02; 54 year-old, 25 years of experience].

*“Perhaps the government and MCPG, can jointly negotiate with the suppliers on the cost of each medicine involved. This will be the medication cost that the supplier will supply to community pharmacies. Without this mechanism of negotiation involving the government, community pharmacies and professional society, the cost of medicines will be very high for patients.”* [CP08; 69 year-old, 41 years of experience].

*“Maybe the government can have a drug store and process the medicines procurement, where we shall get the medicine stock from. I proposed the government to have such pooled procurement system.”* [CP11; 63 year-old, 36 years of experience].

##### 3.2.3.6 Drug costs and professional fees

Community pharmacists emphasised that appropriate remuneration is essential for providing repeat medicines supply services. They expected compensation through professional dispensing fees, medicine cost reimbursement, or a combination of both, recognising the professional effort involved as well as the additional operational costs associated with the service.

Pharmacists also highlighted that the service would increase workload, staffing needs, inventory holding, and financial risk due to medicine expiry and uncertain patient uptake. These factors were perceived to create significant financial exposure for community pharmacies.

*“A dispensing fee will help to cover the additional manpower costs incurred in the community pharmacy because there’ll be more walk-ins and more work to be done. So, if there’s a dispensing fee then that it would be good.”* [CP05; 35 year-old, 14 years of experience].

*“I have to stock up more medicines and don’t know whether the patients will gonna show up. The medicines have the expiry dates if they are expired, who is going to be responsible, right? So we are taking a risk by participating in this project.”* [CP04; 41 year-old, 18 years of experience].

In terms of remuneration structure, pharmacists suggested standardized fee models such as per-service fees, per-medicine charges, or time-based professional fees. Some also proposed regulated medicine pricing and reimbursement mechanisms, including fixed mark-ups and flexibility in generic substitution to ensure cost control and service sustainability.

Collectively, pharmacists stressed that a structured and regulated remuneration framework is necessary to ensure financial viability and long-term participation of community pharmacies in repeat medicine supply services.

*“Depending on how many medicines are in a prescription, maybe there is a minimum fee of RM5 per script, but if there’re more than 5 types of medicines, prescription fees may be increased accordingly. So, I am suggesting a minimum of RM5 per script or trip.”* [CP01; 55 year-old, 25 years of experience].

*“It’s not just about medicines procurement and dispensing, we also need to think about the storage, definitely it will involve costs. So, the markup of 5% helps with the costs of handling, logistic and storage.”* [CP06; 41 year-old, 12 years of experience].

*“Let the community pharmacists have the flexibility to choose which brand they are going to supply, but with a pre-determined medicine cost reimbursement. For example, the government will only reimburse community pharmacists 10 cents per tablet for this medicine, we just have to source it of any brand with less than 10 cents, if not then it isn’t beneficial to pharmacy.”* [CP10; 53 year-old, 22 years of experience].

Pharmacists further raised concerns about sustainable programme financing, suggesting that funding could potentially come from government sources or private insurance providers. Additionally, drawing from previous partnership experiences, they highlighted the risk of delayed reimbursement payments, which could negatively affect pharmacy cash flow and disrupt daily operations.

*“Reimbursement from previous programmes was delayed, sometimes up to six months, despite supplier credit terms of 30–60 days, creating significant cash flow pressure for pharmacies. I remember that was a bad, horrible experience.”* [CP03; 46 year-old, 21 years of experience].

These financial challenges were perceived to have broader implications for programme sustainability, as delayed and unpredictable reimbursement may discourage long-term participation by community pharmacies and threaten the viability of repeat medicine supply initiatives.

#### 3.2.4 Theme 3: community pharmacists’ motivation to participate

Overall, most pharmacists expressed support for involving community pharmacies in supplying repeat medicines to patients with chronic diseases from public health facilities. Their willingness was largely driven by the perceived benefits to patients and the healthcare system, particularly in improving patient care and medication access. For independent and smaller pharmacy chains, the collaboration was also viewed as an opportunity to enhance business sustainability in a highly competitive market. Additionally, pharmacists noted that such a programme would allow them to fully utilise their professional training as healthcare providers and could represent a step towards the long-discussed separation of prescribing and dispensing in Malaysia.

*“It’s definitely beneficial, particularly for improving medication compliance and accessibility for patients. This partnership would be the right way forward.”* [CP05; 35 year-old, 14 years of experience].

*“I face strong competition from chain pharmacies, with fewer walk-in customers. This collaboration could help sustain my income and keep the pharmacy operating.”* [CP14; 52 year-old, 20 years of experience]

*“I think this could be the best chance to practice what I learned. With patient’s walks in with prescription, I just do what I learned to do and we are not talking about prices anymore.”*

However, pharmacists also identified several factors that could discourage participation. These included potential financial risks, logistical and operational challenges, and limited digitalisation of processes, and excessive administrative workload that might detract from patient care. Some pharmacists also noted that the business models of certain corporate-owned pharmacy chains may not be aligned with serving the broader patient demographics expected under such a programme.

*“If the deal is not fair but money-losing, why would I want to join, right? And have more burden on me, I wouldn’t join.”* [CP04; 41 year-old, 18 years of experience].

*“If I have to collect the medicines from the hospital and it’s very far from where I practice. Logistic issues.”* [CP06; 41 year-old, 12 years of experience].

Although these concerns were raised, they should be interpreted alongside previously identified facilitators such as perceived patient benefits, improved healthcare access, and opportunities for professional role enhancement, suggesting that participation decisions are shaped by a balance between perceived benefits and operational constraints.

## 4 Discussion

This study explored Malaysian community pharmacists’ perspectives on participating in a public–private collaboration to supply repeat medicines to patients with chronic diseases from public health facilities using a mixed-methods approach. The quantitative findings indicated that pharmacists strongly supported the proposal, particularly recognizing its potential to improve patient convenience, time flexibility, and accessibility of medicines. They also identified timely reimbursement, access to patient information through EMR, and pharmacist remuneration as key facilitators, while generally not perceiving lack of skills or knowledge as barriers. Most pharmacists expressed willingness to participate in the collaboration and considered RM4.10–RM5.00 per occasion to be a reasonable remuneration. The qualitative findings complemented these results by revealing broader perceived benefits for patients, community pharmacies, and public healthcare facilities, while also identifying additional implementation considerations such as medicine procurement, programme financing, and operational mechanisms.

One of the most prominent findings from both the quantitative and qualitative components was the perceived improvement in patient convenience and access to care. Pharmacists believed that allowing patients to collect repeat medicines from community pharmacies closer to their homes or workplaces would reduce travel distance, transportation costs, and waiting time at public health facilities. Improved convenience was also expected to benefit caregivers who often collect medicines on behalf of patients. These factors may enhance medication adherence and reduce the likelihood of missed medicine refills. Community pharmacies are well positioned to support such services, as there are more than 3,500 community pharmacies nationwide (48), many of which operate with extended hours and do not require appointments. This accessibility positions community pharmacists as some of the most readily available healthcare providers in the community.

Beyond convenience, qualitative findings indicated that community pharmacists viewed their role in repeat medicines supply as extending beyond dispensing to include comprehensive pharmaceutical care, such as medication review, counselling, and promoting medication adherence. This aligns with evidence that community pharmacists in Malaysia are already actively engaged in chronic disease management services. A nationwide study involving 420 community pharmacists reported that almost all pharmacists provided health screening and monitoring for chronic diseases, while 68.8% conducted routine medication reviews in their premises (11). Although large-scale Malaysian studies examining outcomes of pharmacist-led medication review are limited, a pilot study by Karuppannan et al. (2017), (49) demonstrated positive patient outcomes following medication reviews conducted by community pharmacists, with patients requesting continuation of the service. Nevertheless, effective medication review requires timely access to patient health information, and access to EMR was therefore identified as an essential facilitator by pharmacists in this study. Similar observations have been reported in other healthcare systems where access to patient data supports safer medication management and improved care coordination (50).

The perceived benefits of the collaboration were not limited to patients but also extended to public healthcare facilities. Pharmacists believed that redirecting repeat medicine dispensing to community pharmacies could reduce congestion and workload at public health facilities, allowing hospital pharmacists to focus more on clinical pharmacy services and patient counselling. This shift could improve the overall efficiency of healthcare delivery and potentially enhance patient outcomes. With community pharmacists supporting patient self-care education and adherence monitoring, there is also potential to improve chronic disease management and reduce complications. Although Malaysian evidence on the impact of community pharmacists on hospital readmission rates remains limited, international evidence suggests that pharmacist involvement in post-discharge care can reduce hospital readmissions (51).

Community pharmacists themselves also perceived benefits from such collaboration. Malaysian community pharmacists have previously been reported as underutilised within the healthcare system (8). Participation in repeat medicines supply programmes could provide them with opportunities to serve a wider patient population and strengthen recognition of their role as healthcare providers. In the context of Malaysia’s highly competitive community pharmacy market (52), increased patient visits may also contribute to the sustainability of community pharmacy businesses. Additionally, pharmacists viewed such collaboration as a potential step towards the broader policy discussion surrounding the separation of prescribing and dispensing, which is not currently implemented in Malaysia.

Successful implementation of the programme would require a clear regulatory and operational framework. Both quantitative and qualitative findings highlighted the need for a SOP outlining service processes, responsibilities, and professional service requirements for community pharmacists. The Ministry of Health could consider adopting approaches similar to the Omnibus Budget Reconciliation Act of 1990 (OBRA’90) in the United States, which mandates pharmacist counselling and medication review to improve medication safety and reduce healthcare costs (53). Accreditation requirements for participating pharmacies may also be necessary to ensure adequate infrastructure, pharmacist availability, waiting areas, and information systems. The SOP should also specify aspects such as medicine procurement mechanisms, formulary management, and generic substitution policies.

Although pharmacists did not perceive lack of competency as a major barrier, most respondents indicated that additional training would be beneficial for ensuring high-quality service delivery. This finding reflects the positive professional attitude of Malaysian community pharmacists towards continuous professional development, as previously observed by Alakhali et al. (2021), (54). At the same time, pharmacists emphasised the importance of fair remuneration to compensate for increased operational costs associated with the programme. These costs may include additional staffing, inventory management, infrastructure adjustments, and service delivery time. Ensuring sustainable financial mechanisms is therefore crucial for programme success. Professional bodies, pharmaceutical suppliers, and the Malaysian MOH should collaborate with community pharmacists to establish appropriate funding and reimbursement structures. A pharmacoeconomic evaluation comparing current repeat medicine supply practices with the proposed model would also be valuable in estimating the potential impact on national healthcare expenditure.

Pharmacists’ previous experiences with earlier public–private collaborations, such as programmes supplying medicines to government pensioners, also highlighted potential challenges. These include concerns about delayed reimbursement payments, uncontrolled medicine costs, and involvement of third-party entities that may raise confidentiality or conflict-of-interest issues. Addressing these concerns through transparent governance mechanisms and timely reimbursement systems will be essential for building trust among community pharmacists and ensuring programme sustainability.

Future research should explore the perspectives of patients receiving care from public health facilities, as their acceptance of collecting repeat medicines from community pharmacies will be critical to programme success. In addition, a pilot implementation study involving selected community pharmacies and public healthcare facilities would be valuable to evaluate the operational feasibility, cost implications, and patient outcomes of such collaboration.

## 5 Study limitations

This study has several strengths. By employing a mixed-methods design, it provides a comprehensive understanding of community pharmacists’ perspectives through both quantitative breadth and qualitative depth. The nationwide survey captured responses from a large number of pharmacists, while the interviews provided contextual insights into implementation considerations that were not captured in the survey. However, several limitations should be acknowledged. First, the response rate of the survey was relatively modest, which may introduce response bias. Second, the qualitative findings were based on a limited number of interviews, although data saturation was achieved. Third, the study focused on pharmacists’ perspectives and did not include views from other stakeholders such as physicians, policymakers, or patients. Lastly, although measures were implemented to minimise duplicate responses by disabling the “multiple responses” feature in SurveyMonkey®, this approach cannot fully prevent participants from submitting multiple entries using different devices, browsers, or email addresses. Despite these limitations, the findings offer valuable insights for policymakers and healthcare planners considering the integration of community pharmacies into repeat medicine supply services.

## 6 Conclusion

This study examined the perspectives of community pharmacists in Malaysia regarding their participation in a public–private collaboration to supply repeat medicines to patients with chronic diseases from public health facilities. The findings indicate strong support among community pharmacists for such an initiative, with most recognising its potential to improve patient convenience, access to care, and medication adherence, while also reducing congestion and workload in public healthcare facilities. Pharmacists also expressed readiness to contribute beyond medication dispensing by providing comprehensive pharmaceutical care, including counselling, medication review, and chronic disease monitoring. Key facilitators identified included timely reimbursement, access to patient information through electronic medical records, clear operational guidelines, and fair remuneration, whereas operational and financial uncertainties were highlighted as important considerations for successful implementation. Overall, the study suggests that community pharmacists represent a valuable yet underutilized resource within Malaysia’s healthcare system. Future efforts should focus on developing clear policy frameworks, establishing sustainable financing mechanisms, and conducting pilot programmes and patient acceptance studies to evaluate the feasibility and impact of integrating community pharmacies into repeat medicines supply services.

## Data Availability

The original contributions presented in the study are included in the article/Supplementary material, further inquiries can be directed to the corresponding author.
