# Supplementary material for: Enhancing chronic disease medicine access through public–private collaboration: insights from community pharmacists
Source: Front Public Health. 2026 Jun 4;14:1839372. doi: 10.3389/fpubh.2026.1839372 (PMC13275434; doi:10.3389/fpubh.2026.1839372)
Supplement: Supplementary file 1 [file Table_1.DOCX]

Data Collection Form - Quantitative Study

**Inclusive Criteria**

This study is for Fully-Registered Pharmacists (employed, employer or shareholders) working full-time in community pharmacies whether at the pharmacy outlet, warehouse, procurement, marketing or administrative departments.

This study is NOT for Provisional-Registered Pharmacists (PRP) and part-time employed or locum pharmacists.

1. Are you a Fully-Registered Pharmacist working full-time in a community pharmacy?

[ ] Yes

[ ] No

**Section A: Perspectives of Pharmacists from Community Pharmacies**

Patients with chronic diseases usually require multiple medications on a long-term basis. However, medications are often supplied on monthly basis to patients from public health facilities and patients will then be given an appointment for the next visit for their repeat medicines (refill prescriptions), with some opt for getting their supply via mail.

2. We would like to know your opinion if these patients get their first supply of medication from the public health facilities, and subsequently get the repeat medicines from the community pharmacy until their next appointment with the doctor.

For each statement below, please select the answer on a scale from Strongly Disagree to Strongly Agree that best represents what you think.

|  | Question | Strong Disagree | Disagree | Neutral | Agree | Strongly Agree |
| --- | --- | --- | --- | --- | --- | --- |
| i. | I think collecting repeat medicines community pharmacies would save much of the patient's time. | 1 | 2 | 3 | 4 | 5 |
| ii. | I think collecting repeat medicines from community pharmacies would save much of the patient's transportation cost. | 1 | 2 | 3 | 4 | 5 |
| iii. | I think a community pharmacy is a suitable place to provide this service. | 1 | 2 | 3 | 4 | 5 |
| iv. | I think collecting repeat medicines from community pharmacies is convenient for patients in terms of time, due to longer operation hours at community pharmacies. | 1 | 2 | 3 | 4 | 5 |
| v. | I think collecting repeat medicines from community pharmacies of patients' choice is convenient for them in terms of the location. | 1 | 2 | 3 | 4 | 5 |
| vi. | I think community pharmacists should be remunerated for dispensing repeat medicines | 1 | 2 | 3 | 4 | 5 |

3. How likely is your pharmacy to participate in supplying repeat medicines to patients with chronic diseases from public health facilities?

Please select the answer on a scale from Not Very Likely to Very Likely that best represents what you think.

| Not Very Likely | Somewhat Unlikely | Neutral | Somewhat Likely | Very Likely |
| --- | --- | --- | --- | --- |
| 1 | 2 | 3 | 4 | 5 |

4. If there is a dispensing fee for community pharmacists in supplying repeat medicines to patients from public facilities, how much do you think will be a reasonable remuneration for you to provide the service (per occasion)?

[ ] I am willing to provide the service free of charge.

[ ] RM 1.00 or less

[ ] RM 1.10-RM 2.00

[ ] RM 2.10 - RM 3.00

[ ] RM 3.10 - RM 4.00

[ ] RM 4.10 - RM 5.00

[ ] More than RM 5.00 (please specify): ______________

**Section B: Perceived Barriers and Facilitators in Supplying Repeat Medicines**

5. We would like to know your views on potential BARRIERS for community pharmacist AT YOUR PHARMACY to supply repeat medicines to patients from public health facilities.

For each statement below, please select the answer on a scale from Strongly Disagree to Strongly Agree that best represents what you think.

|  | Question | Strong Disagree | Disagree | Neutral | Agree | Strongly Agree |
| --- | --- | --- | --- | --- | --- | --- |
| i. | Lack of time for the community pharmacist to provide the service. | 1 | 2 | 3 | 4 | 5 |
| ii. | Lack of space in my pharmacy for patients' privacy for medication counselling. | 1 | 2 | 3 | 4 | 5 |
| iii. | Lack of space in my pharmacy for a waiting area for patients. | 1 | 2 | 3 | 4 | 5 |
| iv. | Lack of skill or knowledge of community pharmacists to provide the service. | 1 | 2 | 3 | 4 | 5 |
| v. | Lack of confidence of community pharmacists to provide the service. | 1 | 2 | 3 | 4 | 5 |
| vi. | Shortage of community pharmacist in my pharmacy to provide the service. | 1 | 2 | 3 | 4 | 5 |
| vii. | Increased workload for the community pharmacist. | 1 | 2 | 3 | 4 | 5 |
| viii. | Increased operating costs for my pharmacy. | 1 | 2 | 3 | 4 | 5 |

6. What other potential barriers (apart from those mentioned above) that you have in mind? (optional)

Answer:_______________________________________

7. Please rate the importance of having the following FACILITATORS for the new role of supplying repeat medicines for patients with chronic disease from public health facilities.

For each statement below, please select the answer on a scale from Not At All Important to Very Important that best represents what you think.

|  | Question | Not At All Important | Slightly Important | Important | Fair Important | Very Important |
| --- | --- | --- | --- | --- | --- | --- |
| i. | A standard guideline on required services to be provided to the patients. | 1 | 2 | 3 | 4 | 5 |
| ii. | Timely access to patient information through electronic medical records. | 1 | 2 | 3 | 4 | 5 |
| iii. | Timely payment from government to the community pharmacy if it involves reimbursement of medicine cost. | 1 | 2 | 3 | 4 | 5 |
| iv. | Close collaboration with the public facilities in solving patients' medication issues when they arise. | 1 | 2 | 3 | 4 | 5 |
| v. | Community pharmacists’ remuneration for the service. | 1 | 2 | 3 | 4 | 5 |
| vi. | Designated patient waiting area in the community pharmacy. | 1 | 2 | 3 | 4 | 5 |
| vii. | Accreditation for the community pharmacy. | 1 | 2 | 3 | 4 | 5 |
| viii. | Additional training for the community pharmacist. | 1 | 2 | 3 | 4 | 5 |
| ix. | Pharmacy zoning for even community pharmacy distribution. | 1 | 2 | 3 | 4 | 5 |

8. What other facilitators (apart from those mentioned above) that you think are Fairly or Very Important? (optional)

Answer: ______________________________________________

**Section C: Demographic Data**

9. Year of birth:..................

10. Gender:

[ ] Female [ ] Male

11. Ethnic:

[ ] Malay [ ] Chinese [ ] Indian [ ] Others (please specify:_____________)

12. Country of your Degree in Pharmacy?

[ ] Local [ ] Overseas (please specify:____________)

13. Highest level of education:

[ ] Degree in Pharmacy

[ ] Postgraduate Master in Pharmacy

[ ] Postgraduate PhD in Pharmacy

[ ] Others (please state.....................................................)

14. Location of your pharmacy:

[ ] Johor [ ] Penang

[ ] Kedah [ ] Perak

[ ] Kelantan [ ] Perlis

[ ] Kuala Lumpur [ ] Putrajaya

[ ] Labuan [ ] Sabah

[ ] Malacca [ ] Sarawak

[ ] Negeri Sembilan [ ] Selangor

[ ] Pahang [ ] Terengganu

15. Is your community pharmacy participating in the Projek Rintis (Pilot Project) Penyumberluaran (Outsourcing) Bekalan Ubat Susulan ke Farmasi Komuniti (UBAT@Komuniti) by Ministry of Health?

[ ] Yes

[ ] No

16. Job Position:

[ ] Manager [ ] Non-manager

17. Type of Employment:

[ ] Full-time employed pharmacist

[ ] Employer or shareholder pharmacist

18. Department or Job Scope (can tick more than one if relevant):

[ ] Retail at Outlet

[ ] Wholesales at Outlet or Headquarters

[ ] Purchaser at Headquarters

[ ] Marketing at Headquarters

[ ] Administration at Headquarters

[ ] Others (please state:.........................)

19. Number of years working in the community pharmacy in Malaysia (years) :............
