# Supplementary material for: Enhancing chronic disease medicine access through public–private collaboration: insights from community pharmacists
Source: Front Public Health. 2026 Jun 4;14:1839372. doi: 10.3389/fpubh.2026.1839372 (PMC13275434; doi:10.3389/fpubh.2026.1839372)
Supplement: Supplementary file 2 [file Table_2.DOCX]

Interview Topic Guide - Qualitative Study

**Inclusive Criteria**

1. Can you briefly describe your background, role and responsibilities in the company?

**Section A: Perspectives**

2. What do you think, if the government would like to engage the community pharmacists to supply the repeat medicines to patients with chronic diseases from government hospitals/ health clinics? This mean that the patients would get their first newly prescribed medicines from the government hospital/clinic, and then subsequently going to the community pharmacy to collect their repeat medicines until their next visit to their doctors.

Probe:

2i. What made you think so?

2i. If participant mentioned of the benefits or drawbacks: In what way? Please explain.

2ii. If participant mentioned of the effects to one party: How about to others if have?

**Section B: Facilitators**

3. What factors do you think should be in place for such collaboration?

Probe:

3i. If these factors were not mentioned: How about factors such as community pharmacist's capabilities / pharmacy's operating hours / pharmacy distribution / source and availability of the repeat medicines / medicine costs reimbursement?

3ii. If remuneration was mentioned: How much and can you provide the justification?

**Section C: Challenges**

4. What challenges do you foresee from such collaboration?

Probe:

4i. What made you think so?

4ii. Is there anything you can suggest to overcome such challenges?

**Section D: Experience in public-private collaboration**

5. Such collaboration would be a form of Public-private partnership (PPP). Do you have any experience of any PPP before?

Probe:

5i. If yes: Please tell me more about it.

5ii. Is there anything you learned or experienced from the previous PPP, that we can adopt or avoid in this repeat medicine supply by community pharmacists?

**Section E: Willingness to participate**

6. If you are given an option to be involved in such repeat medicines supply, will you choose to collaborate in this public-private partnership?

Probe:

6i. What leads you to this decision?

6ii. What factors could be in placed to change your mind on this decision of yours?

7. Before we conclude today's interview, do you have anything to add?
